# Brazilian Green Propolis: Effects *In Vitro* and *In Vivo* on *Trypanosoma cruzi*

**DOI:** 10.1093/ecam/nep014

**Published:** 2011-02-13

**Authors:** Kelly Salomão, Eniuce M. de Souza, Andrea Henriques-Pons, Helene S. Barbosa, Solange L. de Castro

**Affiliations:** ^1^Laboratório de Biologia Celular, Instituto Oswaldo Cruz, Fundação Oswaldo Cruz, Av. Brasil 4365, Manguinhos 21045-900, Rio de Janeiro, Brazil; ^2^Laboratório de Biologia Estrutural, Instituto Oswaldo Cruz, FIOCRUZ, CP 926, 21045-900, Rio de Janeiro, Brazil

## Abstract

The composition of a Brazilian green propolis ethanolic extract (Et-Bra) and its effect on *Trypanosoma cruzi* trypomastigotes and other pathogenic microorganisms have already been reported. Here, we further investigated Et-Bra targets in *T. cruzi* and its effect on experimental infection of mice. The IC_50_/4 days for inhibition of amastigote proliferation was 8.5 ± 1.8 **μ**g mL^−1^, with no damage to the host cells. In epimastigotes Et-Bra induced alterations in reservosomes, Golgi complex and mitochondrion. These effects were confirmed by flow cytometry analysis. In trypomastigotes, Et-Bra led to the loss of plasma membrane integrity. The *in vitro* studies indicate that Et-Bra interferes in the functionality of the plasma membrane in trypomastigotes and of reservosomes and mitochondrion in epimastigotes. Acutely infected mice were treated orally with Et-Bra and the parasitemia, mortality and GPT, GOT, CK and urea levels were monitored. The extract (25–300 mg kg^−1^ body weight/day for 10 days) reduced the parasitemia, although not at significant levels; increased the survival of the animals and did not induce any hepatic, muscular lesion or renal toxicity. Since Et-Bra was not toxic to the animals, it could be assayed in combination with other drugs. Et-Bra could be a potential metacyclogenesis blocker, considering its effect on reservosomes, which are an important energy source during parasite differentiation.

## 1. Introduction

*Trypanosoma cruzi* is the etiologic agent of Chagas disease, an endemic parasitosis in Latin America infecting 16–18 million people [1]. Acute infections are usually asymptomatic, but the ensuing chronic *T. cruzi* infections have been associated with high ratios of morbidity and mortality. At present, the only accepted drugs for Chagas disease are nifurtimox (Lampit) and benznidazole (Rochagan or Radanil), effective for acute infections, but their use for chronic infections is controversial due to the undesirable side effects, frequently forcing the abandonment of treatment; the poor indices of apparent cure and a lack consensus concerning criteria for parasitological cure. Consequently, the development of alternative drugs for these nitroderivatives is urgent [2, 3].

Although traditional medicines are used worldwide it is often difficult to establish how effective they really are. Only considering parasitic diseases, the examples of quinine and artemisinin suggest that herbal medicines can be very effective medically, and underline the fact that natural products are important sources of new pharmaceuticals. In this context, our laboratory is involved and has been for several years, in the investigation of the effect of propolis on *T. cruzi*, using samples with an established chemical composition [4–6]. Propolis, a product from bees, is extensively used in folk medicine for a wide spectrum of diseases. This resinous material is collected from different plant exudates and thus presents a complex composition depending basically on the plant sources available. Over the last few decades, an increasing number of studies have been published on the chemical composition, biological and pharmacological activities and therapeutic uses of propolis [7]. In temperate zones, poplar trees (*Populus* spp.) are the main source of propolis, while in tropical regions, there are a variety of plant sources, leading to samples with totally distinct compositions [8]. Different animal models have been used to investigate propolis as an anti-inflammatory [9], cariostatic [10] and anti-parasitic agent [11] and its protective role in models of carcinogenesis [12] and hepatotoxicity [13]. Such effects have been associated with the presence of flavonoids, aromatic acids and esters and their anti-oxidative properties [14]. Propolis extracts present low toxicity to experimental animals and humans [15]; in mice the LD_50_ being higher than 7 g kg^−1^ [16] and the dose of 1400 mg kg^−1^ body weight/day for 90 days was proposed as a NOEL (no-effect level) [17].

Due to its characteristics, Brazilian propolis has been the subject of intensive research over the last few decades. It has been sub-divided into four types based on the association of ethanol extracts of Brazilian samples with the levels of bioactive compounds [18]. The subtype BRP1 corresponds to the Brazilian green propolis produced in Southeastern Brazil and its main botanic source is *Baccharis dracunculifolia* (Asteraceae). Green propolis is highly recommended by modern herbalists since it displays microbicidal, anti-inflammatory, immunomodulatory and anti-ulcer properties [19]. We previously determined the chemical composition, the analgesic and anti-inflammatory activities and the *in vitro* and *in vivo* effect on *T. cruzi* of a standard ethanol extract of a Bulgarian sample [4, 6, 20]. Our group also compared its activity with that of a Brazilian green propolis extract (Et-Bra) on *T. cruzi* and on different species of *Leishmania* as well as other pathogenic microorganisms [5, 21]. Giving continuity to the study with Et-Bra, we are presently investigating potential targets in *T. cruzi* using electron microscopy and flow cytometry techniques and also its effect on the course of experimental acute infection in mice.

## 2. Materials and Methods

### 2.1. Propolis Sample and Preparation of the Extract

The Brazilian green propolis sample was collected at Mar de Espanha (State of Minas Gerais, Brazil) from a native forest with a predominance of *B. dracunculifolia*. After cooling below −10°C the resin was cut in small pieces and extracted with 70% ethanol for 24 h as previously described [5] and the residue was named Et-Bra.

### 2.2. Parasites

We used the Y strain of *T. cruzi*. Epimastigote forms were maintained in LIT medium supplemented with 10% fetal calf serum (FCS) and harvested during the exponential phase of growth. Bloodstream trypomastigotes were obtained from infected albino Swiss mice. Amastigotes were collected from the supernatant of trypomastigote-infected J-774G-8 macrophage lineage [22].

### 2.3. Effect on *T. cruzi* Proliferation

Stock solution of Et-Bra was prepared in dimethylsulfoxide (Merck, Darmstadt, Germany). Epimastigotes were resuspended in LIT medium to a parasite concentration of 10 × 10^6^ cells mL^−1^. This suspension (500 *μ*L) was added to an equal volume of the propolis extract, previously prepared at twice the desired concentrations in the same medium in 24-well plates and then incubated at 28°C. For tissue culture derived-amastigotes, the protocol was similar: the experiments were performed at 37°C in DME plus 10% FCS (DMES) in 96-well plates (Nunc Inc., Naperville, IL, USA) [23]. Cell counts were performed after 1 day for amastigotes and up to 4 days for epimastigotes. Et-Bra was assayed in the range of 3–1000 *μ*g mL^−1^ and the final concentration of the solvent never exceeded 0.5%, which had no deleterious effect on the parasites. The activity of the extracts was expressed as IC_50_ values, corresponding to the concentration that leads to 50% inhibition of parasite proliferation.

Peritoneal macrophages were obtained from Swiss mice and were plated in 24-well plates (3 × 10^5^ cells/well) and after 24 h were infected with trypomastigotes (10 : 1 parasite : host cell) in DMES. After interaction for 3 h, the cultures were washed to remove non-interiorized parasites and Et-Bra was added in a final concentration ranging from 15 to 60 *μ*g mL^−1^. At specified intervals the cultures were fixed in Bouin's solution, stained with Giemsa and counted, using the percent of infection and the number of parasites/infected cell as parameters. Host cell viability was measured by the MTT assay [24].

### 2.4. Ultrastructural Analysis

Epimastigotes and trypomastigotes (5 × 10^6^ cells mL^−1^) were treated for 24 h with Et-Bra at concentrations below the corresponding IC_50_ values. For trypomastigotes this value was 66.2 ± 3.7 *μ*g mL^−1^ [5]. Treated and controls parasites were processed for ultrastructural analysis. For scanning electron microscopy, the parasites were adhered to poly-l-lysine-coated coverslips, fixed with 2.5% glutaraldehyde in 0.1 M Na-cacodylate buffer (pH 7.2) at room temperature for 40 min and post-fixed with a solution of 1% OsO_4_, 0.8% potassium ferricyanide and 2.5 mM CaCl_2_ in the same buffer for 30 min. The cells were dehydrated in an ascending acetone series, dried by the critical point method with CO_2_ (CPD 030, Balzers, Switzerland). The samples were mounted with silver cellotape on aluminum stubs, coated with a 20 nm thick gold layer and examined in a 940 DSM Zeiss microscope (Oberkochen, Germany). For transmission electron microscopy, after washing in PBS, the parasites were fixed, post-fixed and dehydrated as described above and embedded in epoxy resin. Ultrathin sections (Leica Ultracuts, UCT, Vienna, Austria) were stained with uranyl acetate and lead citrate and then examined in a EM10C Zeiss microscope (Oberkochen, Germany).

### 2.5. Analysis of the Mitochondrion and Acidic Compartments

After the same treatment described for the ultrastructural analysis (see above), epimastigotes or trypomastigotes were incubated for 15 min with 10 *μ*g mL^−1^ propidium iodide (PI) plus 10 *μ*g mL^−1^ rhodamine 123 (Rh123), or else with 10 *μ*g mL^−1^ acridine orange (AO). Samples were kept on ice until acquisition using a FACSCalibur flow cytometer (Becton-Dickinson, CA, USA). The acquisition was performed in FL1 to Rh123, FL2 to AO and FL3 to PI. Analysis was performed using the Cell Quest software (Joseph Trotter, Scripps Research Institute, San Diego, CA, USA). Control and treated parasites were analyzed using a large region (R1) delimited in FSCXSSC dot plot that could include control parasites and morphological alterations induced by the drug.

Epimastigotes treated with Et-Bra and labeled with AO as described above were washed in PBS and adhered to poli-l-lysine-coated glass coverslips and mounted in DABCO. The material was immediately analyzed and photographed using epifluorescence microscope (Axioplan, Zeiss).

### 2.6. In Vivo Studies

Male albino Swiss mice (age 6–8 weeks; weight 18–20 g) were maintained in our animal facilities in stable conditions of temperature and with 12 h light/dark cycles. Mice were separated into the following groups, each with 8–10 animals: N (non-infected and non-treated), N-PrX (non-infected and treated with Et-Bra at X mg kg^−1^), Tc (infected and non-treated) and Tc-PrX (infected and treated with Et-Bra at X mg kg^−1^). The doses of the extract (X) used were 25, 50, 100, 150, 200 or 300 mg kg^−1^. Groups Tc and Tc-PrX were infected with 10^4^ bloodstream trypomastigotes via the intraperitoneal route (ip). Propolis was administered by gavage for 10 consecutive days beginning on first day post infection (dpi). The body weight was monitored each week. The level of parasitemia was checked by the Pizzi-Brener method [25]. Briefly, 5 *μ*L of blood is added between a slide and a coverslip and 50 fields are counted randomly and the concentration of the parasites was calculated based on a specific factor for a given microscope. The mortality was noted daily and the percent indices of cumulative mortality (%CM) at 40 dpi and the day when mortality attained 50% (M_50_) were calculated. At different points during treatment blood was collected and immediately submitted to analysis for biochemical determination of glutamic oxalacetic transaminase (GOT), glutamate pyruvate transaminase (GPT), urea and total creatine kinase (CK) using the Reflotron System (Roche Diagnostics, F. Hoffmann-La Roche Ltd, Basel, Switzerland). All procedures were carried out in accordance with the guidelines established by the FIOCRUZ Committee of Ethics for the Use of Animals (protocol 0099/01) and by the Guidelines on the Care and Use of Animals for Experimental Purposes (NACLAR).

### 2.7. Statistical Analysis

The comparison between the values of fluorescence for Rh123, PI and AO was performed by ANOVA, followed by the Student-Newman-Keuls test. Statistical significance (*P* < .05) for the *in vivo* experiments was evaluated using the Student's *t* or ANOVA test for the parasitemia and the log rank (Mantel-Cox) test for survival analysis. Kruskall-Wallis and Mann-Whitney tests were used for comparison of GPT, GOT, urea and CK levels among the different experimental groups.

## 3. Results

### 3.1. In Vivo Effect

**Figure 1:**
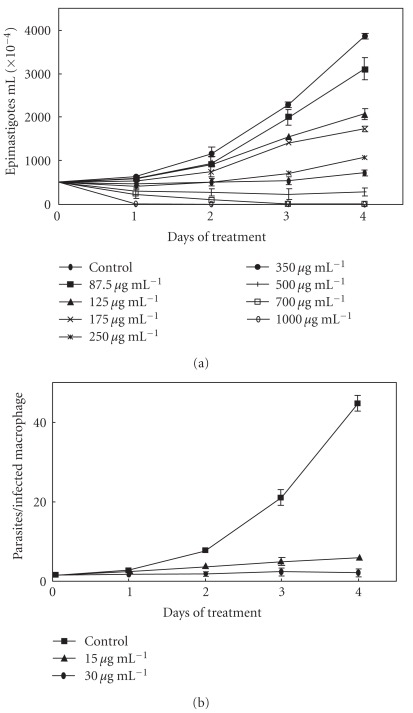
Kinetics of the effect of Et-Bra on *T. cruzi* proliferation. (a) Epimastigote forms. (b) Amastigotes interiorized in peritoneal macrophages.

Et-Bra caused a dose-dependent inhibition of *T. cruzi* proliferation monitored up to 4 days of treatment (Figure 1), intracellular amastigotes (macrophages) being much more susceptible than epimastigotes (9–19-fold). In assays with tissue culture-derived amastigotes, the value of IC_50_/1 day was 18.7 ± 3.2 *μ*g mL^−1^. The IC_50_ values for inhibition of proliferation are displayed in Table 1. The IC_50_ for the inhibition of macrophage infection was: 28.1 ± 3.7, 16.1 ± 8.3, 13.5 ± 3.1 and 15.1 ± 0.2 *μ*g mL^−1^ for 1, 2, 3 and 4 days of treatment, respectively. Damage to the host cells, determined by the MTT assay, was observed only at concentrations higher than 60 *μ*g mL^−1^ Et-Bra.

### 3.2. Ultrastructural Effect

**Figure 2:**
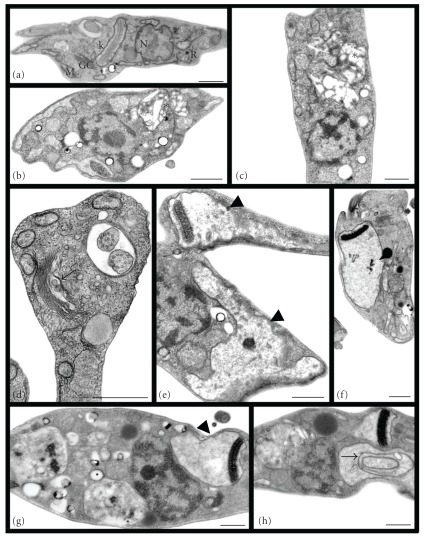
Transmission electron microscopy of *T. cruzi* epimastigotes treated with Et-Bra for 24 h. (a) Control parasite showing the typical elongated body and normal morphology of mitochondrion (M), Golgi complex (GC), nucleus (N) reservosomes (R), and kinetoplast (k); (b) 50 *μ*g mL^−1^ and (c) 100 *μ*g mL^−1^ induced alterations in the morphology of reservosomes (asterisks), with increase of the organelle volume and the number of lipid inclusions and decrease of proteic matrix and vacuolization; (d) 100 *μ*g mL^−1^ induced dilatation of the Golgi complex cisternae (arrow); (e) 100 *μ*g mL^−1^, (f) 250 *μ*g mL^−1^ and (g, h) 300 *μ*g mL^−1^ caused swelling of the mitochondrion with scarcity of the matrix and mitochondrial cristae (arrowheads), with formation of membrane structures inside the organelle (arrow). Bars: 0.5 *μ*m.

**Figure 3:**
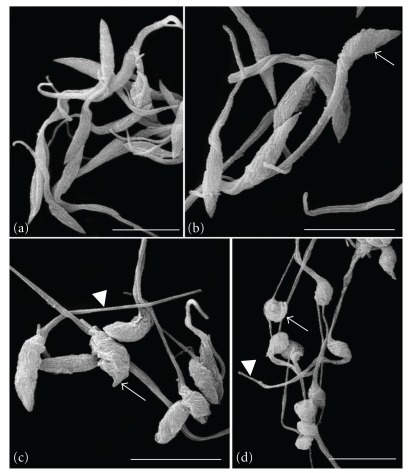
Scanning electron microscopy of *T. cruzi* epimastigotes treated with Et-Bra for 24 h: (a) control parasite with normal morphology; (b) 100 *μ*g mL^−1^, (c) 250 *μ*g mL^−1^ and (d) 400 *μ*g mL^−1^ led to gradual alteration on the morphology, from body shortening to rounded parasites (arrow) with preservation of the flagellum (arrowheads). Bars: 4 *μ*m.

**Figure 4:**
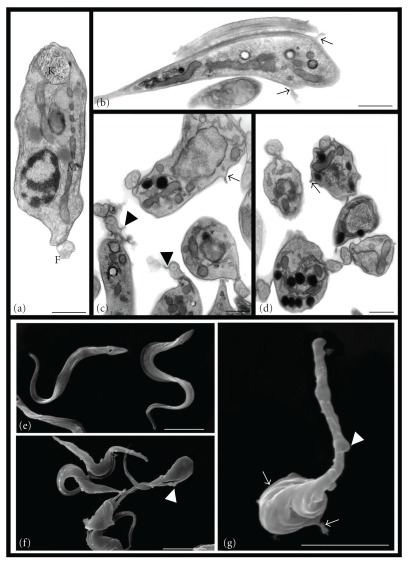
Transmission electron microscopy (a–d) and scanning electron microscopy (e–g) of *T. cruzi* trypomastigotes treated with Et-Bra for 24 h: (a) control parasite with the normal morphology of mitochondrion (M), kinetoplast (k) and flagellum (f); (b) 30 *μ*g mL^−1^ and (c, d) 60 *μ*g mL^−1^ induced the formation of blebs on the body (arrow) and flagellar (arrowheads) membranes. Bars: 0.5 *μ*m. (e) control parasite with the normal morphology; (f, g) 60 *μ*g mL^−1^ leading formation of blebs on the body (arrow) and flagellar (arrowheads) membranes and some rounded parasites (small arrow). Bars: 4 *μ*m.

Epimastigotes and trypomastigotes were treated for 24 h with Et-Bra and analyzed by transmission (TEM) and scanning electron microscopy (SEM) (Figures 2, 3 and 4). In epimastigotes, the extract caused disorganization of the reservosome morphology (Figures 2(b) and 2(c)), revealed by the reduction of matrix electron density and the increase of both its volume and number of lipid droplets, inducing a heterogeneous arrangement of the organelle and the formation of a crystalloid structure (Figure 2(b)); dilatation of the Golgi complex cisternae (Figure 2(d)); mitochondrial swelling with scarcity of matrix and cristae and the presence of membrane structures inside the organelle (Figures 2(e)–2(h)). By SEM, alterations in epimastigote morphology, shortening and rounding of the parasite's body, (Figures 3(b)–3(d)) were observed. Above 400 *μ*g mL^−1^, Et-Bra induced an intense vacuolization, preventing the identification of intracellular organelles and leading to the loss of the typical morphology of epimastigotes observed by TEM and SEM (data not shown). Treatment of trypomastigotes caused irregular expansions (blebs) of the body and flagellar membranes (Figures 4(b)–4(d)), confirmed by SEM analysis (Figures 4(f)–4(g)).

### 3.3. Flow Cytometry Analysis

**Figure 5:**
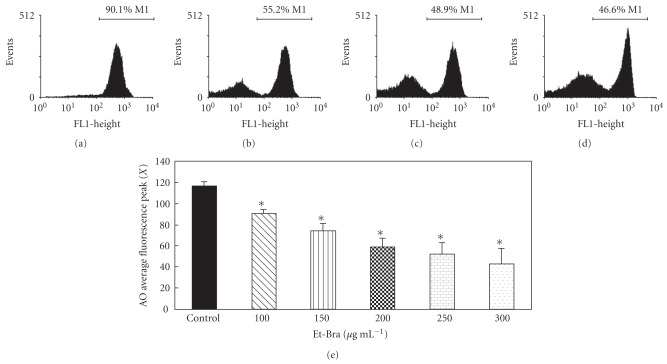
Flow cytometry analysis of *T. cruzi* epimastigotes treated with Et-Bra for 24 h and incubated with Rh123 and PI or AO. (a–d) Rh123 fluorescence intensity in: (a) control, (b) 100 *μ*g mL^−1^, (c) 150 *μ*g mL^−1^, (d) 200 *μ*g mL^−1^ Et-Bra. Rh123^high^ parasites were delimited by a marker (M1) and the corresponding percent of the cells included. (e) Average fluorescence peak values for the AO fluorescence, from at least three independent assays, showing a dose-dependent decrease after treatment with Et-Bra. Asterisks indicate *P* < .05 when compared to untreated epimastigotes.

**Figure 6:**
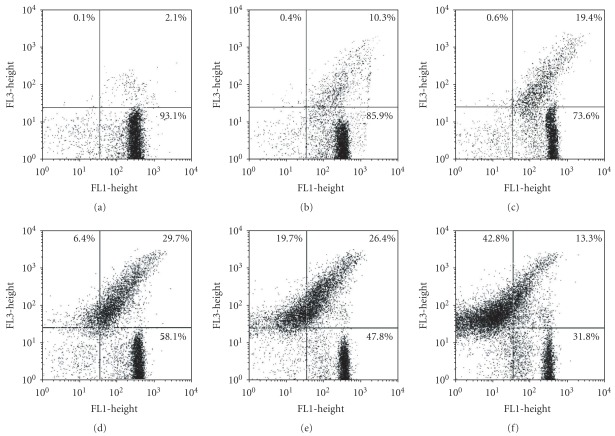
Flow cytometry analysis of *T. cruzi* trypomastigotes treated with Et-Bra for 24 h and incubated with Rh123 and PI. (a–f) Dot plot of parasites labeled with Rh123 (*X* axis) and PI (*Y* axis) and percentages are shown in each quadrant. (a) Control, (b) 15 *μ*g mL^−1^, (c) 30 *μ*g mL^−1^, (d) 60 *μ*g mL^−1^, (e) 100 *μ*g mL^−1^ and (f) 150 *μ*g mL^−1^ Et-Bra.

Based on the ultrastructural data, treated parasites were incubated with fluorescent markers for flow cytometry analysis. FSCXSSC dot plots show that epimastigotes treated with Et-Bra suffered dramatic morphological alterations (data not shown). The extract induced a decrease in the percentage of Rh123^+^ cells from 87.7 (control) to 43.5 (300 *μ*g mL^−1^ Et-Bra), indicating a loss of the mitochondrial membrane potential, although with membrane integrity, as indicated by PI analysis (Figures 5(b)–5(d)). AO labeling indicated, in comparison with untreated epimastigotes, a dose-dependent and statistically significant decrease in the average fluorescence peak at FL2 induced by Et-Bra (Figure 5(e)), a phenomenon also observed by epifluorescence microscopy (data not shown), indicating pH increase in the acidic compartments. In trypomastigotes, increasing doses of Et-Bra (15–150 *μ*g mL^−1^) led to membrane damage, revealed by a dose-dependent increase in PI labeling and decreased fluorescence of Rh123^high^ (Figure 6).

### 3.4. In Vivo Effect

**Figure 7:**
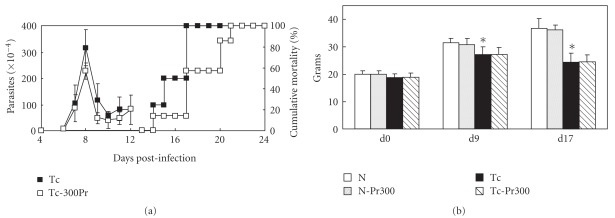
Effect of Et-Bra treatment on *T. cruzi*-infected in mice: (a) parasitemia and cumulative mortality curves of infected mice (Tc) and infected and treated with 300 *μ*g mL^−1^ Et-Bra by oral route, during 10 consecutive days. (b) The body weight of the four experimental groups at days 0, 9 and 17. Asterisks indicate *P* < .05 for comparison of groups N and Tc.

**Figure 8:**
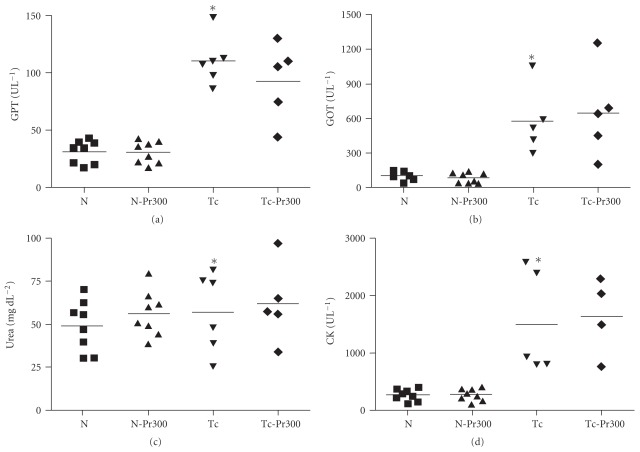
Blood levels of the biochemical markers between non-infected (N), non-infected and treated with 300 *μ*g mL^−1^ Et-Bra (N-Pr300), infected (Tc), and infected and treated with 300 *μ*g mL^−1^ Et-Bra (Tc-Pr300) groups after 14 days. (a) Glutamic oxalacetic transaminase (GOT), (b) glutamate pyruvate transaminase (GPT), (c) urea, (d) creatine kinase (CK). The values were obtained from three independent assays. Asterisks indicate *P* < .05 between groups N and Tc. No statistical difference was observed when treated with the extract.

In relation to *in vivo* experiments, the treatment with 25–300 mg kg^−1^ Et-Bra body weight for 10 consecutive days led to statistically significant decrease in the mortality in comparison with control group, while no important differences were detected in the parasitemia curve (Table 2 and Figure 7(a)). In relation to body weight, the values of infected and treated groups (Tc-PrX) were similar to those of the group Tc (infected and non-treated), being both significantly lower than that of the two non-infected groups (N and N-PrX) (Figure 7(b)). In all the experimental groups at day 14 the GPT, GOT, urea and CK levels were measured directly in the blood. Comparing groups N and Tc, it was observed that infection by *T. cruzi* led to a significant increase in the four markers: 4.0× for GPT, 8.8× for GOT, 1.3× for urea and 3.6× for CK. Administration of Et-Bra at 300 mg kg^−1^ led to similar levels of GPT, GOT, urea and CK between the groups N and N-Pr300, indicating that the treatment of the animals with the extract induced no toxicity, as judged by these parameters, as well as by their general behavior, also no difference was observed between the two infected groups, untreated (Tc) and treated (Tc-Pr300) (Figure 8 and Table 3).

## 4. Discussion

Propolis presents a complex composition depending basically on the plant sources accessible to the bees. Brazilian samples present striking differences in their chemical composition when compared with samples from temperate zones [8]. Besides, differences are also found among tropical samples depending on the local flora at the site of collection [26, 27]. The sample employed in the present work is a green propolis collected in the State of Minas Gerais, classified as a BRP1 [18], due to the high content of the bioactive compounds 3,5-diprenyl-4-hydroxycinnamic acid, 2,2-dimethyl-8-prenyl-2H-1-benzopyran-6-propenoic acid, 3-prenyl-4-hydroxycinnamic acid, *p*-coumaric acid and the absence of 2,2-dimethyl-6-carboxyethenyl-2H-1-benzopyran.

The effect of Et-Bra was investigated on both proliferative forms of *T. cruzi*. Intracellular amastigotes, which are of clinical relevance, are much more susceptible to the extract than epimastigotes. Alterations in the host cell were observed only at a concentration four times higher than that needed to interfere with parasite proliferation. The effect of the extract was also much more pronounced on trypomastigotes, the non-multiplicative form responsible for the transmission of Chagas disease from the invertebrate to the vertebrate host. The IC_50_ for trypomastigotes is 66.2 ± 3.7 *μ*g mL^−1^ while for benznidazole, the standard drug, this value is 2.8 ± 0.1 *μ*g mL^−1^ [5].

The ultrastructural analysis of epimastigotes treated with Et-Bra showed morphological alterations in the reservosomes, Golgi complex and mitochondrion. Reservosomes are endocytic compartments found in *T. cruzi* epimastigotes and the storage site of endocytosed macromolecules and lysosomal enzymes. They are bounded by a membrane unit, with an electron-dense proteic matrix and electron-lucent lipid inclusions [28]. Similar alterations in reservosomes and mitochondrion were described for a standard ethanol extract of a Bulgarian sample, named Et-Blg [6]. Since reservosomes are crucial for the storage of lipids and proteins in epimastigotes [29], a decrease in their electrondensity suggests interference of Et-Bra with the accumulation of ingested macromolecules. Alterations in the Golgi complex induced by the extract could compromise the glucosylation and/or protein transport from the endoplasmic reticulum to secretory vacuoles and lysosomes. Labeling with AO revealed, by epifluorescence, acidic vesicles turning to neutral ones (yellow to green). Such alteration could be due to a reduced activity of reservosome H^+^ pump caused by membrane damage and the consequent efflux of H^+^ leading to pH equalization with the cytoplasm. The increase of lipid accumulation and the lower matrix electrondensity in reservosomes, together with the decrease of AO fluorescence and structural alterations of the Golgi suggest that Et-Bra interferes with the endocytic pathway. The reduction of the electrondensity by propolis could be due to a lower content of proteins, essential for the metacyclogenesis to trypomastigotes [30]. The presence of crystalloid structures inside reservosomes associated with an increased amount of lipids were previously described as differentiated lipid domains [31]. The increase of the total lipid/protein rate observed in epimastigotes treated with Et-Bra reinforces the idea of parasite metabolism alterations leading to its death. Mitochondrial damage is an alteration usually observed in parasites incubated with different drugs due to the fundamental role of this organelle on the overall metabolism of *T. cruzi* [32]. The ultrastructural damage caused by Et-Bra on the mitochondrion was confirmed by the flow cytometry experiments. At concentrations below the IC_50_ value, Et-Bra led to a statistically significant decrease in the percent of Rh123^high^, indicating, interference with the mitochondrial membrane hydrogenionic potential. In Et-Bra-treated trypomastigotes alterations of the plasma membrane were observed by transmission and scanning electron microscopy, suggesting that propolis interferes with membrane fluidity affecting its functionality.

Taken together, these data indicated that, in epimastigotes, mitochondria and reservosomes are target organelles for propolis. In treated trypomastigotes, plasma membrane damage, interference with the mitochondrion function and loss of parasite viability were observed. Interestingly no major mitochondrial damage was detected by TEM; while by flow cytometry a significative decrease of Rh123^high^ labeling was determined, suggesting that a functional alteration precedes a structural one. How propolis acts has not been fully clarified, but in the present study we reported for the first time that the treatment of *T. cruzi* with propolis interferes with the mitochondrial membrane potential in both forms of the parasite and with acidic compartments in epimastigotes.

In continuity, we analyzed the effect of Et-Bra on *T. cruzi*-infected mice. The mice infected and not treated with propolis (Tc) exhibited the classical pattern of parasitemia and mortality rate as previously described [25]. The extract induced a non-significant decrease of parasitemia levels when from 25 to 300 mg kg^−1^ Et-Bra was administered from 1 to 10 dpi, causing a significant decrease of mortality, but did not reverse the loss of weight induced by the infection. Treatment with 100 mg kg^−1^ benznidazole induced both a significant decrease of mortality (25%) and of parasitemia [33]. The activity of the hepatic enzymes, GPT and GOT, urea and CK was measured in the non-infected (N) and infected (Tc) groups, to be used as potential markers of *T. cruzi* infection. This approach was feasible only up to 14 dpi, since afterwards the number of animals in Tc group gradually decreased, preventing a reliable comparison. Putting the results of three independent experiments together, the values of the four parameters for Tc were always significantly higher than those of N. However, it is important to note the results obtained with urea and CK. For urea, in two out of three independent experiments no difference was observed between groups N and Tc (*P* = .071 and *P* = .439), while in the third one such difference was significative (*P* = .016). However, when the values of all the experiments were taken together, the difference between these two group was significative (*P* = .004). For CK, the infected group in each experiment always presented higher enzyme levels than in N. Therefore, further work is needed to ascertain if urea and CK are reliable parameters to monitor the course of infection.

The activity of these four biochemical markers was also evaluated in treated groups to evaluate any potential toxicity of Et-Bra (N versus N-PrX) and interference in the course of infection (Tc versus Tc-PrX). Our results show that Et-Bra up to 300 mg kg^−1^ for 10 days by oral route did not induce hepatic (GPT, GOT) and renal toxicity (urea) or led to muscular damage (CK) in non-infected mice. In the Tc-Pr300 group a significant increase in the levels of these four, at levels similar to those of the infected group, (Tc) occurred, indicating no protection or exacerbation of the infection. CK and the isoenzyme from the heart (CK-MB) increase during an acute *T. cruzi* infection with a positive correlation between both enzymes and heart inflammatory infiltration [34]. Both enzymes were measured in the plasma using the commercial kits (Granutest, Merck), after adaptation for low volumes of plasma [34]. In the present work CK was measured directly in the blood and a 3.6-fold increase of enzyme levels was observed in infected mice after 14 days of infection.

Propolis is known as an immunomodulatory agent due to its known effects on antibody production and on different cells of the immune system [35]. In this context, experiments are currently underway in our laboratory to investigate if Et-Bra exerts any influence in the immune response in *T. cruzi*-infected animals. It would also be interesting to investigate the association of propolis with benznidazole. Another aspect that needs to be further analyzed is the potential role of Et-Bra as a metacyclogenesis blocker, due to the alterations in the morphology and physiology of reservosomes here described, since these organelles play a fundamental role in this differentiation process in the insect vector gut.

## Funding

This work was supported by CNPq, DECIT/SCTIE/MS, Papes/FIOCRUZ and FAPERJ.

## Figures and Tables

**Table 1:** Values of IC_50_ (*μ*g mL^−1^) for the effect of Et-Bra on the proliferative forms of *T. cruzi*.

Days of treatment	Epimastigotes	Intracellular amastigotes	Extracellular amastigotes
1	347.8 ± 31.7^a^	38.5 ± 4.6^b^	18.7 ± 3.2
2	234.5 ± 18.2	16.7 ± 3.0	—
3	218.3 ± 26.4	11.3 ± 2.35	—
4	149.0 ± 4.6	8.5 ± 1.8	—

Dash indicates not determined.

^a^Mean ± SD of three independent experiments.

^b^Values based on the number of parasites/infected macrophage.

**Table 2:** Values of the parasitemia peak for the treatment of *T. cruzi*-infected mice with different doses of Et-Bra^a^.

Experiment	Groups	Parasitemia peak (parasites mL^−1^ (×10^−4^))
No. 1	Tc	558.2 ± 209.7
	Tc-Pr25	399.9 ± 150.0
	Tc-Pr50	363.6 ± 151.4

No. 2	Tc	255.6 ± 159.6
	Tc-Pr100	205.2 ± 76.9
	Tc-Pr150	210.0 ± 80.0

No. 3	Tc	326.0 ± 145.9
	Tc-Pr200	255.2 ± 95.6
	Tc-Pr300	227.7 ± 33.4

^a^Treatment for 10 consecutive days beginning at 1 dpi.

**Table 3:** Blood levels of biochemical markers in the experimental groups^a^.

Groups	GPT (U/L)	GOT (U/L)	Urea (mg/dL)	CK (U/L)
N	31.0 ± 10.0	99.8 ± 41.1	48.9 ± 14.7	270.6 ± 101.1
N-Pr300	30.6 ± 9.7	84.7 ± 42.0	56.2 ± 13.2	275.1 ± 105.1
Tc	109.9 ± 20.9^b^	573.6 ± 289.5^b^	57.0 ± 22.9^b^	1507.4 ± 900.8^b^
Tc-Pr300	92.6 ± 33.8	647.6 ± 391.1	61.7 ± 22.0	1641.5 ± 675.8

^a^Deterenation after treatment for 10 consecutive days.

^b^ *P* < .05 between groups N and Tc.
